# Enhanced bactericidal effect of ceftriaxone drug encapsulated in nanostructured lipid carrier against gram-negative *Escherichia coli* bacteria: drug formulation, optimization, and cell culture study

**DOI:** 10.1186/s13756-020-0690-4

**Published:** 2020-02-10

**Authors:** Sahar Ebrahimi, Nafiseh Farhadian, Mohammad Karimi, Mohsen Ebrahimi

**Affiliations:** 1 Chemical Engineering Department, Faculty of Engineering, Ferdowsi University of Mashhad, Mashhad, Iran; 2 Department of Emergency Medicine, Faculty of Medicine, Ahvaz Jundishapur University of Medical Sciences, Ahvaz, Iran; 3 Cardiovascular Research Center, School of Medicine, Mashhad University of Medical Sciences, Mashhad, Iran; 4 Department of Emergency Medicine, Faculty of Medicine, Mashhad University of Medical Sciences, Mashhad, Iran

**Keywords:** Ceftriaxone sodium, Antimicrobial drug resistance, Drug delivery systems, Side effects

## Abstract

**Background:**

Ceftriaxone is one of the most common types of antibiotics used to treat most deadly bacterial infections. One way to alleviate the side effects of medication is to reduce drug consumption by changing the ordinary drug forms into nanostructured forms. In this study, a nanostructured lipid carrier (NLC) containing hydrophilic ceftriaxone sodium drug is developed, and its effect on eliminating gram-negative bacteria *Escherichia coli* death is investigated.

**Methods:**

Double emulsion solvent evaporation method is applied to prepare NLC. Mathematical modeling based on the solubility study is performed to select the best materials for NLC preparation. Haftyzer-Van Krevelen and Hoy’s models are employed for this purpose. Drug release from optimized NLC is examined under in vitro environment. Then, the efficacy of the optimized sample on eliminating gram-negative bacteria *Escherichia coli* is investigated.

**Results:**

Mathematical modeling reveals that both methods are capable of predicting drug encapsulation efficiency trends by chaining solid and liquid lipids. However, Haftyzer-Van Krevelen’s method can precisely predict the particle size trend by changing the surfactant types in water and oily phases of emulsions. The optimal sample has a mean particle size of 86 nm and drug entrapment efficiency of 83%. Also, a controlled drug release in prepared nanostructures over time is observed under in-vitro media. The results regarding the effectiveness of optimized NLC in killing *Escherichia coli* bacteria suggests that by cutting drug dosage of the nanostructured form in half, an effect comparable to that of free drug can be observed at longer times.

**Conclusion:**

Results confirm that NLC structure is an appropriate alternative for the delivery of ceftriaxone drug with a controlled release behavior.

## Introduction

Ceftriaxone sodium is an antibiotic commonly used for the treatment of bacterial infection such as middle ear infection, meningitis, bone, and joint infection, intra-abdominal infection, skin infection, and pelvic inflammatory diseases [1]. Ceftriaxone vials are among the most prevalent types of antibiotics with the highest mortality rate due to the injection of vials [2]. Ceftriaxone produces several side effects such as diarrhea, elevated liver enzymes, blood urea nitrogen, eosinophilia, thrombocytosis, and other local reactions [1]. Given the inevitability of using this antibiotic in today’s health care system, it is essential to develop new prodrugs [1].

Recently, nanoparticle delivery systems have been employed for the encapsulation of lipophilic, hydrophilic, and poorly water-soluble drugs [3]. The use of lipids for the formation of nanoparticles such as solid lipid nanoparticles (SLN) or nanostructured lipid carrier (NLC), offers multiple benefits compared to other materials, which is due to low cytotoxicity and controlled drug release [4]. The controlled drug release is aimed to maintain drug concentration in the blood or the target tissue at the favorable level [5]. The controlled drug release can be applied to both hydrophilic and hydrophobic drugs. For example, Tamoxifen hydrochloride, as a hydrophilic drug, is encapsulated in a microemulsion system with a controlled release. It is effective in the breast cancer treatment in comparison with the commercial forms of the drug [6]. Raloxifene [7] and Clozapine [8], as hydrophobic drugs in microemulsion and nanostructured lipid forms, have exhibited essential growth in the drug release compared to free dugs.

To date, lipid nanoparticles have been successfully used for hydrophobic drug entrapment, though encapsulating a high content of hydrophilic drugs in these materials is challenging. The key parameters in the preparation of NLC containing hydrophilic drugs are formation method, selection of materials as solid and liquid lipids, and choice of appropriate surfactants used in organic and water phases. Double emulsion solvent evaporation is a technique of NLC preparation that involves hydrophilic drugs [9]. However, the best materials for NLC preparation could be determined by mathematical methods. Group-contribution methods such as Hoy [10, 11] and Hoftyzer-van krevelen [12] are excellent candidates for this purpose. These methods have been successfully applied to predict particle size and drug entrapment efficiency [13, 14].

This is the first study to prepare a nanostructured form of ceftriaxone sodium based on NLC using appropriate controlled drug release. We then test the efficacy of this nanostructure on E.coli bacteria, as a highly resistant gram-negative bacteria, and compare results to that of free drugs. Given that particle size and drug entrapment efficiency have a noticeable effect on the bacteria death efficacy, we also investigate the methods of determining the best materials for forming small-size particles with high drug encapsulation efficiency. Mathematical modeling based on group-contribution methods is utilized for this purpose. Various formulations are explored to investigate the effect of lipid types and surfactants on the particle size and drug entrapment efficiency. The accuracy of mathematical prediction is also evaluated by an experimental study. Finally, the best NLC formulation and a more accurate mathematical model are introduced. The main objectives of the study are as follows:

- To determine the accuracy of mathematical modeling based on group-contribution method in predicting the particle size and drug entrapment efficiency.
- To determine the effectiveness of ceftriaxone drug in both free and nanostructured forms in cell culture media.
- To find the best drug dosage in the nanostructured form to achieve comparable antibacterial efficacy in comparison with the free drug.
- To determine rate of bacteria death using NLC form of ceftriaxone drug.

## Mathematical modeling

### Hoftyzer-Van Krevelen’s method

A group-contribution method for predicting the solubility parameter of components is Hoftyzer-Van Krevelen’s method. In this method, the solubility parameter is calculated from the following equations [12]:

1 $$ {\updelta}_{\mathrm{d}}=\frac{\varSigma {F}_{di}}{V_m} $$

2 $$ {\updelta}_{\mathrm{p}}=\frac{\sqrt{\varSigma {F}_{pi}^2}}{V_m} $$

3 $$ {\updelta}_{\mathrm{h}}=\sqrt{\frac{\sum {E}_{hi}}{V_m}} $$

4 $$ {\updelta}_{\mathrm{t}}=\sqrt{\delta_d^2+{\delta}_p^2+{\delta}_h^2} $$

In the above equations, *V*_*m*_ is the molar mass, δ_d_ and δ_p_ are dispersion and polar forces, δ_h_ is the hydrogen bonding of solubility parameters, δ_t_ is the total solubility parameter, *F*_*di*_ and *F*_*pi*_ are dispersion and polarization components in the molar function, respectively, and *E*_*hi*_ is the role of hydrogen bonding force in the tensile energy between molecules.

### Hoy’s method

There is another model for predicting the solubility parameter, the equations of which are as follows [11]:

5 $$ \upalpha \left(\mathrm{p}\right)=777\times {\varDelta}_T^P/\mathrm{V} $$

6 $$ \mathrm{n}=0.5/{\varDelta}_T^P $$

7 $$ {\delta}_t=\frac{F_t+\frac{B}{n}}{V} $$

8 $$ {\updelta}_{\mathrm{p}}={\updelta}_{\mathrm{t}}\left(\frac{1}{\alpha (p)}\frac{F_p}{F_T+\left(\frac{B}{n}\right)}\right)0.5 $$

9 $$ {\updelta}_{\mathrm{h}}={\updelta}_{\mathrm{t}}\left(\frac{\alpha (p)-1}{\alpha (p)}\right)\kern0.37em 0.5 $$

10 $$ {\updelta}_{\mathrm{d}}={\left({\updelta_{\mathrm{t}}}^2+{\updelta_{\mathrm{p}}}^2+{\updelta_{\mathrm{h}}}^2\right)}^{0.5} $$

In these equations, α(p) is the number of molecular aggregates in each component, n is units repeated in each part of the molecular chain and B is a constant (equal to 277) [11]. Also, *F*_*t*_, is the molar attraction function, *F*_*p*_ is its polar components and ${\Delta }_T^P$ is the Lydrsen-Hoy constant.

### Material selection based on mathematical methods

In this study, two important NLC parameters, particle size and drug entrapment efficiency, are investigated. To examine the trend variation of these parameters, it is necessary to calculate the solubility parameters of all components separately, and then compare their numerical values with each other. The chemical structure of substances plays a crucial role in computing the solubility parameter. The solubility parameter of lipid components and drug can influence drug entrapment efficiency, while the solubility parameter of surfactants and lipids may affect the particle size.

Since natural oils are composed of several components, it is not possible to calculate their solubility parameters. Hence, another effective parameter called hydrophilic to hydrophobic balance (HLB) is used for comparison.

The HLB value of pure lipids is calculated by Eq. 11, while HLB of natural oils such as sesame oil, which is a mixture of different components, is obtained from Eq. 12.

11 $$ \mathrm{HLB}=\frac{20}{\ 1+\frac{K}{\left[{\delta_d}^2+(0.25){\delta_p}^2+(0.25){\delta_h}^2\right]}\ } $$

12 $$ \mathrm{HLB}=\frac{W_A\ast {HLB}_A+{W}_B\ast {HLB}_B+{W}_C\ast {HLB}_C+\dots }{W_A+{W}_B+{W}_C+\dots } $$

In Eq. 11, K equals 43 in emulsion systems, δ_d_ and δ_p_ are dispersion and polar forces, respectively, and δ_h_ is the hydrogen bonding of solubility parameters. These parameters are obtained from Haftyzer-van Krevelen and Hoy’s methods. In Eq. 12, W_A,_ W_B,_ and W_C_ represent the weight fraction of components A, B, and C, respectively C [15].

## Experiments

### Materials

Ceftriaxone sodium was purchased from Exir Pharmaceutical Company (Borujerd, Iran). Stearic acid and glycerol mono-stearate as solid lipids, oleic acid as liquid lipids, soy lecithin, Span 80, polyvinyl alcohol (PVA), Tween 80 as surfactants, and ethanol as solvents were purchased from Merck Company. Deionized water was used in all experiments.

### Preparation of NLC

In the first step, 0.3 g of GMS (solid lipid) and 0.09 g of oleic acid (liquid lipid) are mixed with 0.95 ml of ethanol (solvent) and 0.055 g of soy lecithin (oil phase). The resulting mixture is placed in a water bath at 60 °*C*. Then, 0.15 ml deionized water containing 0.2 g/l drug is placed in a water bath (internal water phase). Due to high hydrophilicity of the drug, it dissolves rapidly in deionized water and generates a light yellowish color. We prepare the external water phase containing deionized water and Tween 80 with a concentration of 0.275 g/l and place it in a water bath. Then, the oily solution is homogenized by the ultrasonic probe at 80 rpm for 5 min. Finally, internal and external water phases are added, respectively. After 5 min of ultrasonication, the sample is cooled at 0–4 °C. In the final step, a magnet is inserted inside the sample and it is stirred for 1 h at ambient temperature to evaporate the solvent [16, 17].

### Drug entrapment efficiency

A high-performance liquid chromatography analysis is employed to measure drug loading in the NLC structure. Drug loading efficiency is calculated from the amount of drug not loaded in the structure according to Eq. 13:

13 $$ \mathrm{Drug}\ \mathrm{loading}\ \mathrm{efficiency}\ \left(\%\right)=\frac{Amount\ of\ primer\ drug- amount\ of\ unloaded\ drug\ }{Amount\ of\ primer\ drug}\ast 100 $$

**Fig. 1:**
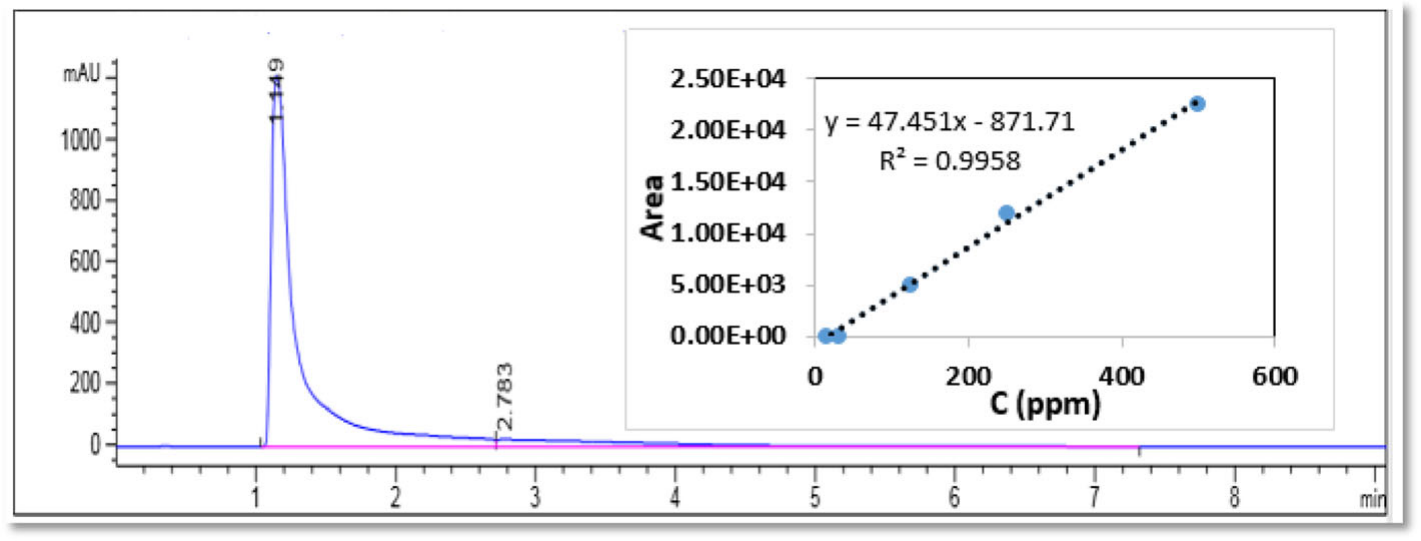
HPLC diagram of ceftriaxone sodium drug at a concentration of 500-ppm (internal diagram is HPLC calibration curve)

Drug loading was calculated by a high performance liquid chromatographic (HPLC) test using the Agilent 1260 machine. We used C18 column for this purpose. The column diameter and length were 4.6 mm and 100 mm, respectively. The injection volume was 50 μl with a flow rate of 1 ml per min at a pressure of 120 psi and the column temperature of 24 °*C*. The mobile phase of ceftriaxone drug detection was containing as 95% methanol and 5% water solution. Drug detection was conducted using a UV-visible detector at a wavelength of λ = 240 μm. Figure 1 shows the HPLC diagram. As can be seen, the drug residence time is 1.149 min.

To calculate the unloaded drug, 5 ml of each sample was centrifuged at 14000 rpm for 20 min at 4 °C. Then, the supernatant solution was transferred from the filter (0.22 μm). After diluting the solution with deionized water with a 1:10 ratio, it was transferred to the chromatography apparatus.

### Characterization

Particle size analysis (PSA) and zeta potential of lipid nanoparticles were measured by light scattering analysis (Vasco 3, Cordouan, France). NLC was also diluted with distilled water to obtain a suitable scattering intensity.

The morphology of nanoparticles was measured by the TEM analysis (Leo 912 AB, American). The surface attributes of the sample were determined by an AFM analyzer (Full model Ara pazhoohesh, Iran). Crystallography of samples was examined by X-ray diffraction (XRD) analysis (Bruker D8 Advance, Germany) for lipid, drug, NLC-enricheddrug, and NLC-free drug. To investigate whether all materials are present inside NLC, we conducted the FT-IR analysis for the above four samples (Thermo Nicolet, American)

### In vitro drug release

To examine drug efficacy in blood-resembling environment, drug release was implemented under in vitro conditions. PBS buffer at pH = 7.4 was selected for this purpose. Five milliliters NLC sample containing ceftriaxone sodium was placed in a dialysis bag (12 kDa). The same volume of suspension with an identical content of free drug was poured separately into a dialysis bag. The bags were then floated in 50 ml buffer for 48 h in an incubator shaker at 37 °C. After 0.5, 1, 2, 6, 8, 24, and 48 h, 1 ml of the sample was removed from the buffer and the same pure buffer was immediately replaced in the system. Samples were sent to the HPLC machine to determine the amount of drug. According to the HPLC calibration curve, the drug content in each sample was calculated. Then, the profile of drug release versus time was plotted for both pure drug and nanostructure samples. It should be noted that the drug content removed at each sampling time was added to the next dose, and the cumulative effect of the drug was considered in calculations.

### Cell culture

In this study, we used the 24-h stratification method for *Escherichia coli* strain ATCC 35218. For this purpose, the bacterial cell culture was performed in a Muller-Hinton agar medium, a suspension with a half-MacFarland equivalent to 0.85% of normal saline. Under these conditions, the number of bacteria is about 1.5*10^8^ CFU/ml. This suspension was used in the next steps of the experiment [18].

Antibiotic solutions, as free and nanostructure forms, were prepared in sterile distilled water freshly during the day.

#### MIC determination

To determine the minimum growth inhibitory concentration (MIC) of ceftriaxone in free and nanostructure forms, the broth dilution method (macro dilution) was used under Clinical and Laboratory Standard Institute (CLSI) guidelines [19]. The antibiotic solution (ceftriaxone sodium) was prepared in pre-sterilized water and preserved at − 70 °C in a frozen state until it was used. In the serial dilution, we used concentrations of 0.0078, 0.0156, 0.0312, 0.0625, 0.125, 0.25, 0.5, 1, 2, 4, 8, 16, 32, 64 and 128 μg/mL and a tube as the positive control (no antibiotics). We prepared a 24-h bacterial culture in a sterile suspension physiological serum equivalent to 0.5 McFarland standard turbidity. The culture was followed by incubation of media containing antibiotics, which were then transferred to 35R oven and remained there for 24 h to grow completely. After 24 h, tubers were examined for ocular hyperactivity as a sign of bacteria growth. Any type of opacity (in a specific or concise manner) was considered as resistance to dilution. The minimum concentration of bacteria without visible growth was considered as MIC [19].

#### Bacterial death kinetics

To plot the bacterial death curve at the specified time, *E. coli* was cultured on a nutrient agar medium at 37 °C for 24 h. In the next step, 3–5 colonies of pure culture were removed and inoculated in Muller Hinton Broth medium containing a suspension equal to 0.5 MacFarland turbidity in normal saline (0.85%). Under these conditions, the number of bacteria was about 1.5*108 CFU / ml. Then, 100 μl of this standard suspension was added to the culture tubes containing 2 ml of Muller Hinton broth medium, which consisted of the free form of ceftriaxone sodium and nanostructured ceftriaxone sodium with various concentrations. The tubes were placed at 35 °C with continuous movement in a shaker incubator. Then, after 2, 4, 6, 8, and 10 h, using 100 μl of each sample, the number of living bacteria was counted using the sequential dilution method [20].

## Results

### Mathematical results

The results of solubility parameters calculated from Hoy and Haftyzer-Van Krevelen’s methods are presented in Table 1.

**Table 1:** Calculated solubility parameters using Hoy and Haftyzer- Van Krevelen methods

Material type	Material name	Solubility Parameter Hoftyzer-van krevelen method	Solubility Parameter Hoy method
Solid lipid	Stearic acid	17.43	18.16
Solid lipid	GMS	19.37	20.93
Liquid lipid	Oleic acid	17.31	18.31
Surfactant	Soy lecithin	18.84	17.42
Surfactant	Span 80	22.48	22.01
Surfactant	PVA	28.98	21,012
Surfactant	Tween 80	19.17	21.86
Drug	Ceftriaxone sodium	32.14	22.09

To study the effect of chaining materials on drug entrapment efficiency, it is necessary to calculate the difference between solubility parameters of drug and lipids. For liquid lipids, the HLB value is examined. Sesame oil is composed of linoleic acid (39–59%), oleic acid (35–54%), palmitic acid (10%) and stearic acid (5%). These values were extracted from our previous study based on GC analysis [21]. Results are shown in Table 2.

**Table 2:** Difference in solubility parameters of various components

Solubility parameters difference (Δδ_t_)			
Drug	Lipid	Haftyzer -van krevelen method	Hoy method
Ceftriaxone sodium	Stearic acid	14.71	4.79
Ceftriaxone sodium	GMS	12.77	2.02
HLB of Solid and Liquid lipids			
Lipid type	Lipid	Haftyzer -van krevelen method	Hoy method
Solid lipid	GMS	17.603	17.569
Solid lipid	Stearic acid	17.328	17.568
Liquid lipid	Oleic acid	17.292	17.564
Liquid lipid components	Linoleic acid	16.880	17.227
	Oleic acid	17.392	17.564
	Palmitic acid	17.473	17.908
	Stearic acid	17.328	17.568
Liquid lipid	Sesame oil	17.146	17.433

As shown in Table 2, in both methods of solubility parameter calculation, the solubility parameter difference in drug-GMS is lower than the drug-stearic acid. It is therefore anticipated that the nanocarrier provided with GMS will have a higher drug content compared to nano-carriers provided with the stearic acid lipid. However, drug molecules are usually solved in liquid lipids, too. The HLB value for various lipids is reported in Table 2.

As shown in Table 2, the amount of HLB associated with lipids is not significantly different, indicating a similar ratio for the hydrophobic and hydrophilic property of these lipids. However, since ceftriaxone sodium has a hydrophilic nature, it dissolves in the hydrophilic lipid. Therefore, based on HLB calculation, the selected solid and liquid lipids will be glycerol mono-stearates (HLB_GMS_ > HLB_SA_) and oleic acid (HLB_oleic acid_ > HLB_sesame oil_), respectively. The entrapment efficiency is predicted based on solid-liquid lipids as follows:

- Stearic acid- sesame oil< Stearic acid- oleic acid< GMS- sesame oil < GMS- oleic acid

The accuracy of the predicted trend can be determined by an experimental data later.

To predict the trend of NLC particle size with various solid and liquid lipids, we investigated the difference between solubility parameters of solid lipids and surfactants (Table 3). A lower difference between solubility parameters of surfactants and solid lipid indicates a smaller nanoparticle size. Since the lipid content of solid lipid is higher than that of liquid lipid, the effect of solid lipid solubility parameter on nanoparticle size is expected to be higher than that of the liquid lipid.

**Table 3:** Difference between lipid solubility parameter and stabilizer

Samples	Solid Lipid	Stabilizer	Δδ_t_ (Hoftyzer-Van krevelen)	Δδ_t_ (Hoy)
S_1_	Stearic acid	Soy lecithin	1.41	0.74
S_2_	Stearic acid	Span 80	5.05	3.85
S_3_	Stearic acid	Tween 80	1.74	3.70
S_4_	Stearic acid	PVA	11.55	2.96
S_5_	GMS	Soy lecithin	0.53	3.51
S_6_	GMS	Span 80	3.11	1.08
S_7_	GMS	Tween 80	0.20	0.93
S_8_	GMS	PVA	9.61	0.19

As depicted in Table 3, based on the Haftyzer-Van krevelen’s method, the solubility parameter of the active surface material for the internal NLC phase is comparable to that of soy lecithin for both solid lipids. Nevertheless, this parameter is predicted by the Hoy’s method in a completely different manner. It introduces Span 80 as the best surfactant for the system containing glycerol mono-stearate and soy lecithin for a stearic acid solid lipid.

With regard to the external water phase surfactant, it is recommended to use Tween 80 in Hoftyzer-van-krevelen’s method and PVA in the Hoy’s method for both solid lipid systems. Besides, in both methods, the solubility parameter difference in the system containing glycerol mono-stearate is lower than the system containing stearic acid lipid.

Finally, as predicted by Hoftyzer-van krevelen’s method, in the system containing glycerol mono-stearate and soy lecithin, Tween 80 has the minimum size, while in the system containing glycerol mono-stearate, span 80 and polyvinyl alcohol have the minimum particle size, as determined by the Hoy’s method.

It should be noted that the effect of external aqueous phase surfactant on decreasing the nanoparticle size is more significant than that of the internal surfactant water phase. The accuracy of mathematical methods can be verified by experimental methods.

### Comparing mathematical and experimental results

The results of nanoparticles prepared with various components are displayed in Table 4. It is worth noting that we used the same amount of materials in all formulations and only changed the material type.

**Table 4:** Experimental results for particle size and drug entrapment efficiency of various formulations

Sample	Solid lipid	Liquid lipid	Surfactant of the internal water phase	Surfactant of the external water phase	Particle size (nm)	Drug loading(%)
S_1_	GMS	Oleic acid	Soy lecithin	Tween 80	86.09	83%
S_2_	GMS	Oleic acid	Span 80	PVA	112.41	80%
S_3_	GMS	Sesame oil	Soy lecithin	Tween 80	101.95	75%
S_4_	GMS	Sesame oil	Span 80	PVA	127.41	73%
S_5_	Stearic acid	Oleic acid	Soy lecithin	Tween 80	202.74	72%
S_6_	Stearic acid	Oleic acid	Span 80	PVA	260.52	69%
S_7_	Stearic acid	Sesame oil	Soy lecithin	Tween 80	306.75	60%
S_8_	Stearic acid	Sesame oil	Span 80	PVA	369.67	58%

As Table 4 shows, according to experimental results, the drug entrapment efficiency can be boosted by chaining solid and liquid lipids as follows:

- stearic acid- sesame oil< stearic acid- oleic acid< GMS- sesame oil < GMS- oleic acid

The comparison of mathematical prediction and experimental results confirms the accuracy of both Haftyzer van-Krevelen and Hoy’s methods.

The trend of particle size change based on experiments is as follows:

- stearic acid- sesame oil> stearic acid- oleic acid> GMS- sesame oil > GMS- oleic acid

According to the mathematical models and Table 3, it is predicted that in the Haftyzer van-Krevelen’s method, the solubility of internal phase surfactants in both solid lipids correspond to soy lecithin solubility parameter. The sample prepared with soy lecithin as the surfactant in the internal water phase has a smaller nanoparticle size. The results of Table 4 corroborate this prediction. However, the prediction of this parameter with the Hoy’s method is completely different. According to the Hoy’s method, Span 80 is the best surfactant for the system containing glycerol mono-stearate and soy lecithin is the best surfactant for the stearic acid lipid. This prediction is inconsistent with experimental results. As far as the aqueous external phase surfactant is concerned, it is recommended to use Tween 80 in Hoftyzer van-krevelen’s method, and Polyvinyl alcohol in the Hoy’s method for both solid lipid systems. According to Table 4, the particle size of the sample with Tween 80 as the external water phase surfactant is smaller, which confirms the prediction of the Hoftyzer-van krevelen’s method.

Finally, the system containing glycerol mono-stearate and oleic acid as the solid and liquid lipid and soy lecithin and Tween 80 as the surfactant of internal and external water phase was chosen as the optimum sample with a small particle size and high drug loading.

### Characterization of optimized structure

**Fig. 2:**
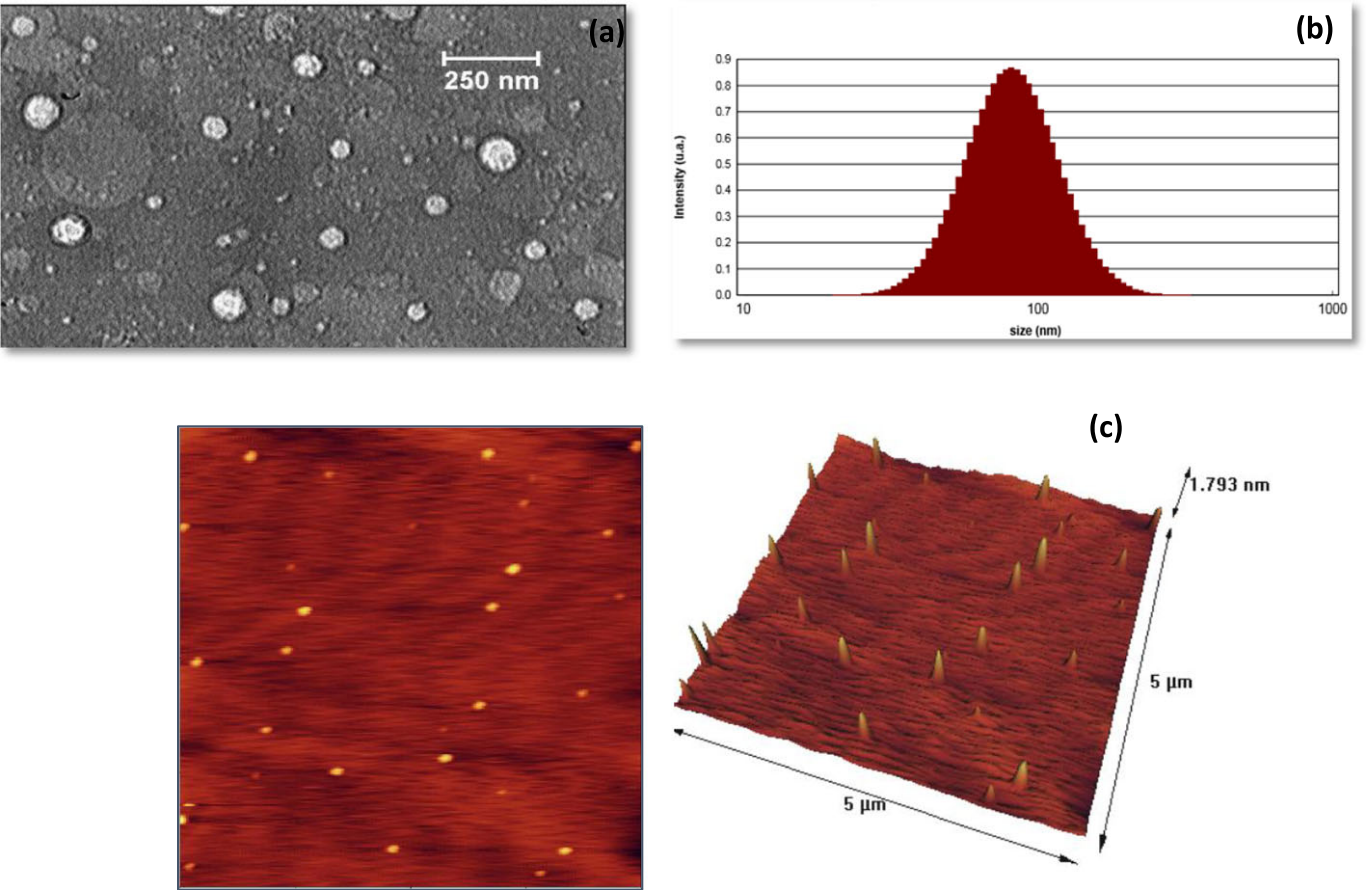
**a** TEM image, **b** PSA image and **c** AFM analysis results at both 2D and 3D view

Characterization was performed following the determination of the optimum sample. Figure 2 shows TEM, PSA and AFM images of the optimum sample. As Fig. 2a illustrates, NLC particles have a spherical structure with a mean particle size of 70 nm, while DLS calculation estimated a particle size of 86 nm (Fig. 2b). TEM and DLS results are relatively identical, and the difference between TEM and DLS analysis is due to the measurement method. The DLS analysis measures the hydrodynamic diameter of particles in the solution, indicating a larger diameter for particles [22, 23]. Moreover, the polydispersity index (PDI) of 0.131 reveals the uniform distribution of particles, which is aligned with AFM images (Fig. 2c). Based on the amount of PDI, values close to zero manifest higher stability of nanoparticles [24]. The zeta potential of the sample is − 20.26, which is acceptable for lipid nanoparticles. This numerical value can be attributed to functional groups with a negative charge on the structure, which generates sufficient repulsion to prevent the agglomeration of particles in the solution in a short time.

**Fig. 3:**
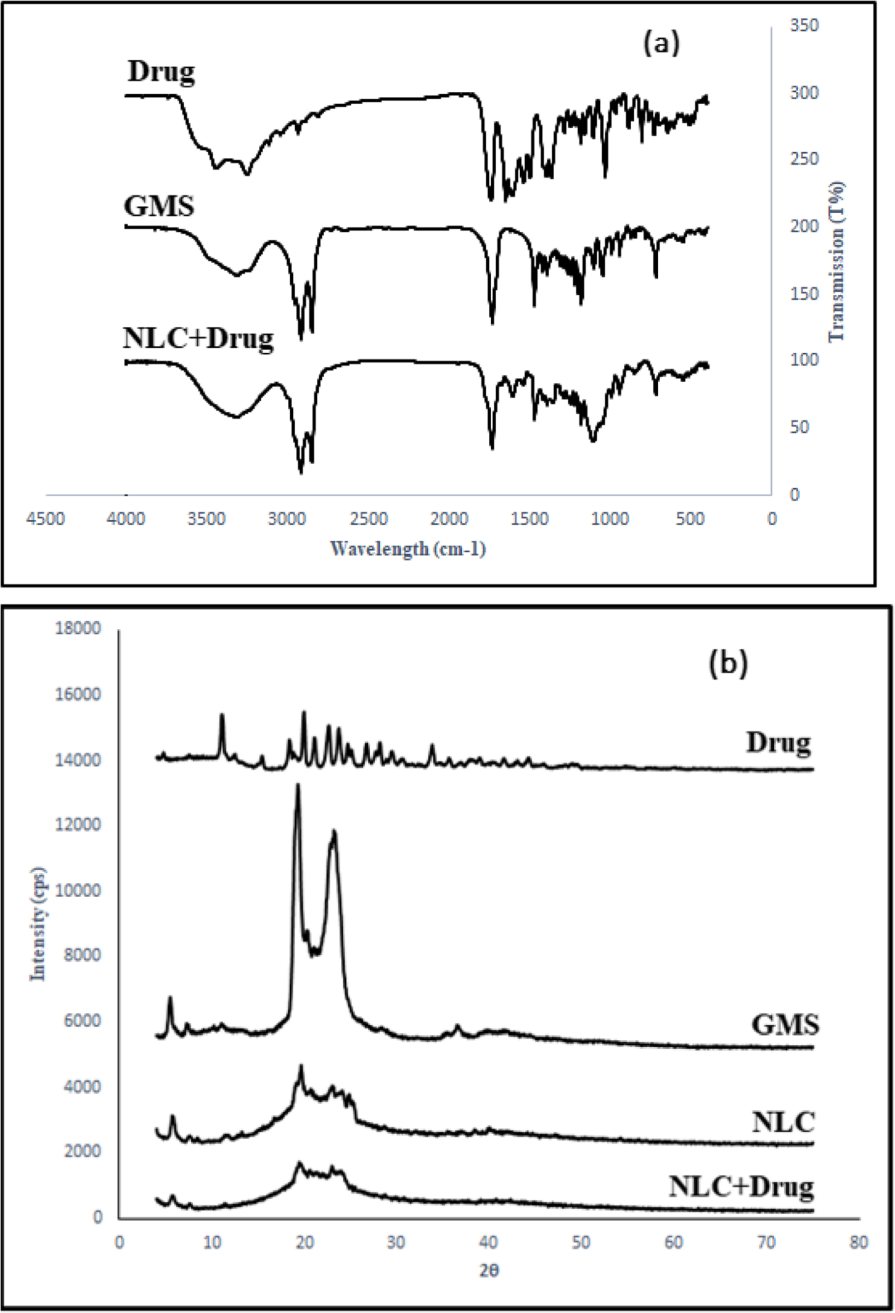
**a** FTIR and **b** XRD analysis

FT-IR analysis was used to examine functional groups on the nano-carrier surface. Figure 3a shows the results of the FT-IR analysis for ceftriaxone sodium, Glycerol Monostearate, and nano-carriers containing ceftriaxone sodium. The FT-IR analysis of ceftriaxone sodium exhibits a band at 3444.63 cm^− 1^, which can be attributed to the stretching vibration of O-H and N-H. The bands appearing at 1747.39 cm^− 1^ and 1649.02 cm^− 1^ are due to the C=O stretching vibrational frequency [25]. Moreover, the bands emerging at 3303.8 cm^− 1^, 1734.8 cm^− 1^, 1179.6 cm^− 1^ are attributable to the O-H bond, C=O bond, and C-O transplant, and bands emerging at 2912 cm^− 1^ and 2846.6 cm^− 1^ are related to the C-H bond of the GMS structure, respectively [26].

As shown in Fig. 3a, the FT-IR spectrum obtained from the NLC sample containing ceftriaxone sodium resembles that of glycerol mono-stearates. This indicates that the drug has been successfully encapsulated in the lipid structure. By comparing the spectra of nanoparticles to each pure component, the presence of all materials can be verified. The peak at 1649.02, which is caused by the C=O amide, confirms drug presence in the NLC structure. Due to low drug content in the nano-structure, the effects of drug-related peaks are weaker.

In the next step, XRD patterns of ceftriaxone sodium, GMS, and NLC with/without drug are measured. According to Fig. 3b, ceftriaxone sodium has sharp edges at 11.18 °, 12.56 °, 18.92 °, 21.24 °, 22.74 °, 23.80 °, 25.20 °, 28.28 ° [27]. Also, glycerol mono-stearate has sharp peaks in the range of 19.97 ° to 23.38 °[28] and glycerol mono-stearate has a crystalline structure. However, as shown by XRD patterns of NLCs containing drug and non-drug, peaks are broader and less sharp than those of pure patterns. These results exhibit the amorphous structure of the prepared NLCs. The changes of crystallography nature can be attributed to the presence of other materials such as surfactants and liquid lipids. Comparing the peaks of pure drug to that of NLC-containing drug reveals that there is no comparable peak in the NLC pattern. This implies that drug has been successfully loaded in the nanocarrier [29, 30].

### In vitro drug release

**Fig. 4:**
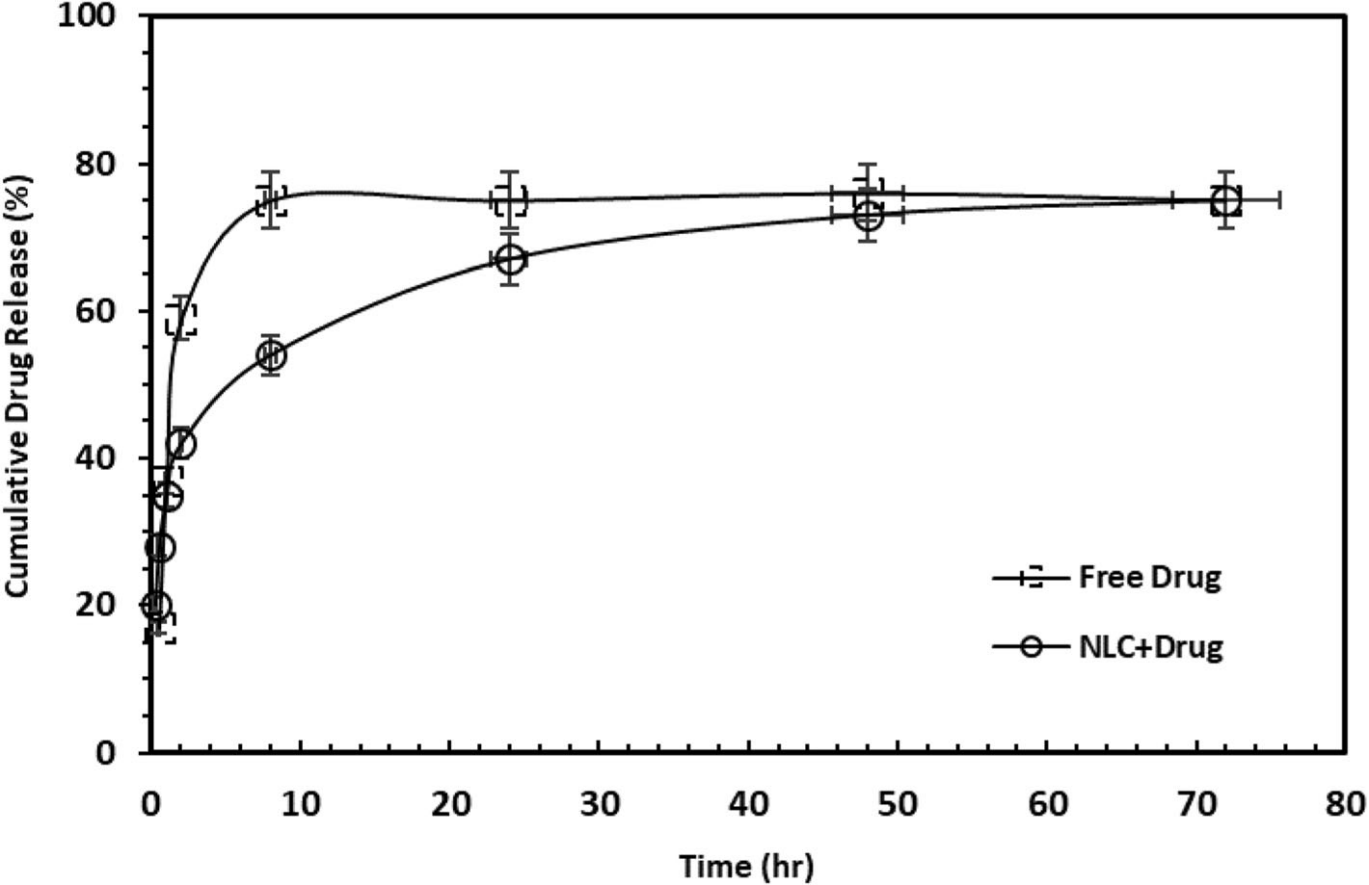
In vitro drug release from free drug and nanostructured NLC form

The graphs in Fig. 4 indicate the release of drug in both commercial and NLC forms. As can be seen, the commercial drug has explosive release while there are two types of drug release in the NLC form: explosive and controlled release. About 42% of the drug is released after 6 h from both formulations. In the case of the commercial market ampoule, the rapid release persists for 10 h and then drug release rate is stabilized (about 80%). However, the gradual release of the nano-carrier sample in this study lasted for 72 h with a moderate gradient. The efficacy of the two drug forms will be further determined by conducting experiments that contain bacterial culture.

### Cell culture results

The results of MIC analysis showed that 2 μg / ml concentration of ceftriaxone in free form and 1 μg / ml concentration of ceftriaxone in nanostructured form inhibited the growth of *Escherichia coli* bacteria.

**Fig. 5:**
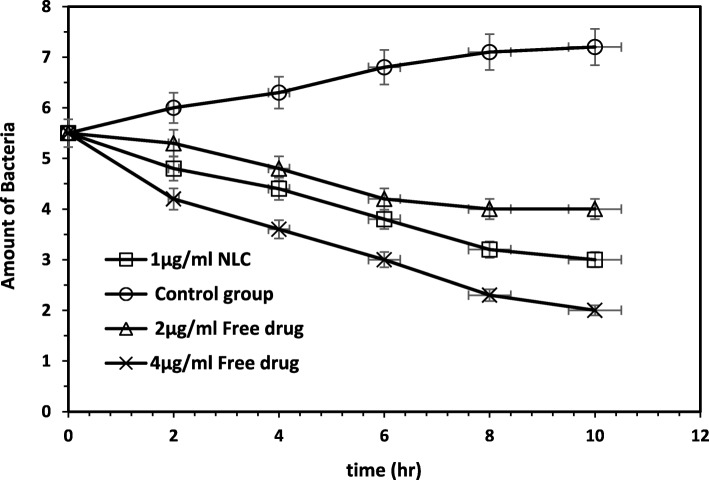
Results of cell culture

The bactericidal activity of ceftriaxone sodium at different concentrations in both free and nanostructure forms was studied by counting bacteria after 0, 2, 4, 6, 8 and 10 h. The results of the death kinetics test are presented in Fig. 5. In this case, we investigated variation in the number of bacteria after 0, 2, 4, 6, 8 and 10 h based on MIC concentration of ceftriaxone sodium in the form of nanostructures under different concentrations of sodium ceftriaxone in free form and antibiotic-free sample.

As Fig. 5 shows, at the same time, the number of bacteria in the system containing the nanostructured form of drug with a concentration of 1 μg/ml is lower than that of a system containing free drug with a concentration of 2 μg/ml.

## Discussion

In this study, a double emulsion solvent evaporation method was used to construct NLC for the delivery of ceftriaxone sodium drug. Hoftyzer-Van Krevelen and Hoy’s methods were applied to calculate solubility parameters. Based on the prediction of two mathematical models and the experimental results, both Hoy and Hoftyzer-van krevelen’s models appear to be strong predictors of drug loading in different NLC systems. However, Haftyzer van-krevelen’s model is capable of predicting the components of NLC to achieve a smaller particle size whereas the Hoy’s method is unable to predict the trend accurately. These results align well with those reported in the literature. Tian et al. demonstrated that the solubility parameter can be a suitable guide for the design and identification of a stable micellar system with a high drug loading capacity [31]. Bahrami et al. argued that solubility parameters based on group-contribution model offer a useful guide for preparing NLC with various formulations containing Fentanyl citrate as the hydrophobic drug. They illustrated that the surfactants-lipids solubility parameter had a bearing on the nanoparticle size, while the drug-lipid solubility parameter affected drug entrapment efficiency [13].

In vitro drug release at pH = 7.4 indicates the controlled release of drug from the structure and the accuracy of the proposed nanostructured behavior. The explosive release of antibiotics from the nanostructure in early hours of injection leads to bacterial death. Then, the gradual release of the nanocarrier sample over time with a mild gradient can diminish the bacterial resistance and enhance drug efficacy. This claim is approved by the cell culture study. The kinetics curve of bacteria mortality indicates that the system containing nanostructure form can last bacterial death for a longer time. Moreover, the slope of curve in the nanostructure sample is more controlled than that of the curve in the free drug. Furthermore, it suggests that by cutting the drug dosage of the nanostructure in half, the same efficacy can be achieved. It should be noted that the permeability of E.coli against antibiotics and antimicrobial agents is low. It is a gram-negative bacterium with a thinner peptidoglycan layer and lipopolysaccharide as the outer membrane. For this reason, *E. coli*’s resistance is higher than bacteria such as *Staphylococcus aureus* [32, 33]. Chaining the common form of the drug to a nanostructure with a controlled drug release can undermine the resistance of the bacteria membrane wall over time and improves the bacterial mortality rate. These observations are aligned with Kumar et al.’s study. They prepared a SLN structure containing Ceftriaxone sodium drug and examined its inhibition effect on *E. coli* bacteria, with their results revealing a slower and sustained release of drug from the SLN structure. Drug release profiles in their study exhibited a sustained release from the SLN structure where only 6% of the drug was released from the nano-formulation in 24 h, whereas 80% of the drug was released for the drug alone [1].

The present study has some strong points worth mentioning. Using mathematical modeling in this study helps overcome some of issues associated with the selection of best materials without experiments. It can affect assessment method and modeling in this field. Moreover, investigating the effect of the NLC form of drug with various doses on *E. coli*, as a highly resistant gram-negative bacterium, is another advantage of this research.

However, there are a number of drawbacks that should be mentioned. In NLC preparation, we changed one parameter and kept others constant. Hence, the inter-variable relationships were not investigated [34]. For the cell culture study, a strict control is required to perform experiments properly. At the same time, a greater degree of control makes experiments artificial. These problems can be solved by performing several experiments to get a clearer picture of the process [35]. Future studies can take measured to address this shortcoming.

A number of limitations should also be noted. First, NLC was prepared using double-emulsion solvent evaporation method by an ultrasonication device. Future studies can utilize other methods and investigate their effects on the particle size and drug entrapment efficiency. Second, we focused on the impact of the NLC form of ceftriaxone on E-coli bacteria in a cell culture study, while the use of nanostructured form of ceftriaxone sodium can have beneficial effects on other types of gram-positive and gram-negative bacteria. A direction of research for future research can be evaluating the impact of nanostructured form of ceftriaxone on various resistant bacteria. Finally, we only measured the effect of the NLC form of the drug under in vitro environment. Future research can conduct similar tests under in vivo media.

## Conclusion

In the present study, the nanostructured lipid carrier was prepared to load ceftriaxone sodium as a hydrophilic drug. The effect of drug on *E. coli* was investigated in bacterial culture media. Based on the mathematical modeling, the Haftyzer-van krevelen’s model appeared to be more accurate than the Hoy’s method in predicting the trend of NLC particle size with different materials. Nevertheless, both Haftyzer-van krevelen and Hoy’s methods were able to predict the trend of change in drug entrapment efficiency accurately. Informed by the kinetics of bacteria cell, the results revealed that the NLC form of drug had higher antibacterial activity against E.coli gram-negative bacteria compared to the free drug. The greater antibacterial effect of the drug at a lower dose in the NLC form is another important finding of this study with regard to antibiotic-dose reduction and cost-effective treatment of resistant microbes.

## Data Availability

The datasets used in the study are available and can be provided by the corresponding author upon request.

## Abbreviations

AFM: Atomic force microscopy
ATCC: American Type Culture Collection
CLSI: Clinical and Laboratory Standard Institute
DLS: Dynamic light scattering
E.Coli: *Escherichia coli* bacteria
FT-IR: Fourier-transform infrared spectroscopy
GC: Gas chromatography
GMS: Glycerol mono-stearate
HLB: Hydrophilic-Lipophilic Balance
HPLC: High performance liquid chromatographic
MIC: Minimum growth inhibitory concentration
NLC: Nanostructure lipid carrier
PDI: Polydispersity index
PSA: Particle size analysis
PVA: Polyvinyl alcohol
SLN: Solid lipid nanoparticle
TEM: Transmission electron microscopy
XRD: X-ray diffraction
